# Mission and challenges of higher education: an interview with G.Q. Max Lu, the president of the University of Surrey

**DOI:** 10.1093/nsr/nwaa072

**Published:** 2020-06-19

**Authors:** Ling Wang, Zhengtang Guo

**Affiliations:** Freelance science writer based in Beijing; Institute of Geology and Geophysics, Chinese Academy of Sciences, and the former vice president of the University of Chinese Academy of Sciences

## Abstract

The University of Surrey (referred to as Surrey hereafter) is one of the renowned universities in the UK that was established on 9 September 1966 with the grant of its Royal Charter and its roots go back to Battersea Polytechnic Institute, founded in 1891. Surrey is the research hub of small satellites, mobile telecommunication and artificial intelligence in Europe. In 2016, Surrey was named as ‘University of the Year’ in the UK and, in February 2018, Surrey won the Queen's Anniversary Prize for Higher and Further Education (Surrey's fourth award)—the highest national award for the UK universities, in recognition of the outstanding contribution of Surrey to nutrition and health.

The president and vice chancellor of Surrey, Professor Max Lu, took this position in 2016 and is also the first scholar of Chinese origin to be the leader of a British university. Before he joined Surrey, he was the provost and senior vice president at the University of Queensland in Australia. Professor Lu is not only a talented leader in education field, but also a distinguished scientist in materials chemistry and nanotechnology area. He has been honored with numerous awards, including the Orica Award, RK Murphy Medal, China International Science and Technology Award and Medal of the Order of Australia, etc. He has been also appointed to the Prime Minister's Council for Science and Technology and the Board of UK Research and Innovation, etc. The rich experience and open-mindedness lead to his profound insights into higher education around the world. Lately elected as a fellow of Royal Academy of Engineering (RAEng) and foreign member of the Chinese Academy of Sciences, Professor Lu shared his broad and deep perspectives on higher education with *National Science Review* during his travel in Beijing.

## THE UNIQUENESS OF SURREY

**NSR:** What kind of university is Surrey?

**Lu:** Surrey is a comprehensive university focusing on engineering, with distinct strengths. Surrey and universities like Huazhong University of Science and Technology and Beihang University in China may have something in common: although the size of the university is not very large, it has clear advantages in some disciplines.

Surrey has expertise and strength in fields such as small satellites, 5G telecommunication, solid-state lasers, artificial intelligence (AI) and autonomous vehicles, intelligent healthcare, Internet of  Things, and block-chain and clean-energy technologies. The emphasis of research at Surrey is not placed on papers or citations, but on its impact on society and the economy.

Since Guildford School of Acting (GSA) became part of Surrey in 2010, which is dedicated to teaching and research in the performing arts, the university has gained more strength in creative-arts disciplines. So far, there have been four Oscar winners, seven Grammy winners and 22 fellows of the Royal Society and Royal Academy of Engineering among Surrey graduates.

**NSR:** Could you describe Surrey using several phrases?

**Lu:** I think the excellent teaching and student experience, high-impact research and unique world-class programs are the most remarkable characteristics of Surrey.

**Figure:**
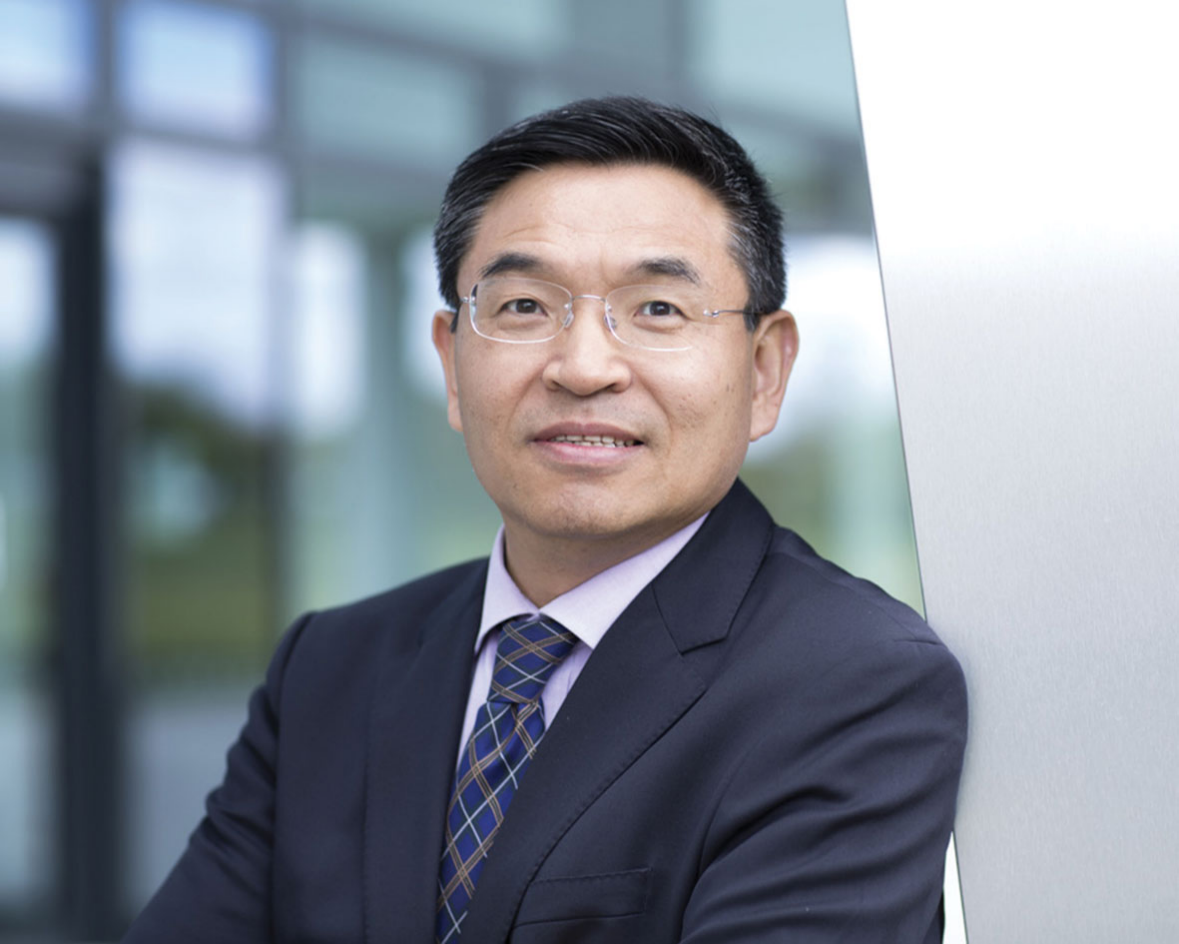
Professor Max Lu is the president and vice chancellor of Surrey, and the first scholar of Chinese origin to be the leader of a British university (*Courtesy of Prof. Max Lu*).

**NSR:** You have mentioned that Surrey places great emphasis on teaching and student experience. Could you give us more details?

**Lu:** The Teaching Excellence Framework is a government assessment of the quality of undergraduate teaching in universities and other higher-education providers in the UK. The framework takes into account students’ satisfaction, achievements, employment rate and salary level as indices. The assessment is implemented every 3 years and the outcomes are characterized by gold, silver and bronze medals, respectively. Surrey won the gold medal in the latest assessment, which is a great recognition of our university's teaching and student experience.

In fact, one of the most important missions of any university is to nurture talent for society. Surrey regards students as both partners and customers of the university, and put the students’ satisfaction and employability at high priority. According to statistics of the UK government in 2014, the employment rate of graduates from Surrey was the highest in the UK (96.9%), which was higher than those of Cambridge University and Oxford University.

Surrey is one of the few universities with a 4-year degree program in the UK. We offer the students options to have a professional training program at government departments, and commercial companies with a salary in their third year of university. Thanks to the internship experience, the students have their confidence and communication skills greatly enhanced and they are better prepared for their career. And that is one of the reasons why the employment rate of Surrey graduates always stays high.

Furthermore, the renowned GSA of Surrey has four graduates who have won Oscars, seven who have won Grammy awards and many have been honored with other prizes. It is a distinctive school with high enrolling standard. Each year, about 3500 candidates go through audition opportunities in the world, only 38 of whom get admitted. The graduates from this school all have multiple offers—even up to 10 offers—when they have graduated and they become leaders of influence in their professions later on.

## RESEARCH EXCELLENCE AT SURREY

**NSR:** Surrey's pioneering small satellites research is highly influential in the world. How does Surrey encourage and support such research ideas?

**Lu:** Exactly. Small satellites is landmark research from Surrey, the idea of which was proposed by Sir Martin Sweeting, a then-PhD candidate in Surrey. It is actually a story about the curiosity and exploration of a scientist. In 1981, Sir Martin Sweeting raised a bold proposal—to produce a small satellite baring all the functions of a traditional satellite but with totally different technologies and at a much lower cost.

Developing and sending satellites were huge projects, always led by national agencies at that time, and Martin's idea was not appreciated by the general public obviously. But he did not give up and finally made the world's first small satellite in 1984 when he became a lecturer at Surrey. To realize his dream, Martin took a step further: asking the president of Surrey for £1 million to launch his satellite. The visionary president of Surrey gave him the budget barely without hesitation, which made his dream come true.

The first small satellite was a thrilling accomplishment and has been changing the landscape of space technology ever since. In 2014, Martin was awarded the Zhao Jiuzhang Medal by the

Chinese Academy of Sciences/International Space Research Committee for his significant contribution to the miniaturization of satellites. His citation reads: ‘Martin used high performance components and devices on the ground to make small satellites with quick response, low cost and high performance, in this way, space mission became possible for less developed countries and even for college students.’

While £17 million were poured into the project, few academic papers had been published during the 8-year project period. But, when it was done, it proved to be the number one in the world.

—Max Lu

**NSR:** What is the latest progress in small satellites?

**Lu:** Surrey keeps on innovating in small satellites and always stays at the forefront. Based on its cutting-edge small-satellite technologies, Surrey Space Science Centre was established in 1979, which later led to the formation of a highly successful spin-off company, Surrey Satellite Technology Ltd (SSTL). SSTL designed and launched a satellite constellation consisting of 22 small satellites that became the first generation of the Galileo global navigation system in Europe.

The REMOVEDEBRIS project led by Surrey Space Science Centre and with the satellite's platform manufactured by SSTL is another example. The project aims to find the best way to capture the estimated 40 000 pieces of space debris orbiting Earth. In June 2018, the satellite named RemoveDebris was finally launched and successfully fulfilled its mission. While £17 million were poured into the project, few academic papers had been published during the 8-year project period. But, when it was done, it proved to be the number one in the world.

Until now, commercial companies like SpaceX, Boeing, Oneweb and Google have all their own plans for sending up a small-satellite constellation that are inseparable from the small-satellite technology invented by Professor Martin Sweeting.

**NSR:** Besides small satellites, the 5G Innovation Centre also plays an important role in advancing wireless-telecommunication technology. What makes the center an innovation hub for 5G technologies?

**Lu:** The 5G Innovation Centre in Surrey is one of the world's first and foremost research centers for forging mobile telecommunication and future internet technologies. The center has attracted about £75 million of investment both from government and from companies like Huawei, British Telecom and many other companies around the world. The center has developed data-transmission technology with a speed reaching 1 Tb/s—more than 100 times faster than that of 4G communication technology.

The 5G Innovation Centre is an R&D institution with close collaboration between academia and industry. There are over 200 researchers at the center, from both Surrey and collaborating enterprises. In 2017, Surrey conducted a pioneering

experiment on 5G technology at our campus, which realized the autonomous car driving on a 5G network. In this project, the Technical University of Munich provided the automobile, Huawei provided the 5G base station and related technologies, and Surrey developed the control system and software. It should be said that only when we have substantial cooperation with enterprises can we efficiently promote the development of the industry.

**NSR:** Research in Surrey is highly interdisciplinary. How do you push research like this?

**Lu:** In order to meet the major challenges of science and society nowadays, expertise from one discipline is not enough and interdisciplinary collaboration is required. For example, materials science is interdisciplinary in nature, covering physics, engineering, chemistry and even medicine. It is just like a kaleidoscope—through different angles, we would be given different combinations. Breakthroughs made in this field are always the result of many brilliant ideas from multiple disciplines converged together.

In Surrey, our vice president for research is responsible for formulating interdisciplinary research policies and developing research programs. The chosen projects will be supported by the university strategic fund and include the involvement of several different research groups.

**NSR:** How would the interdisciplinary projects be selected?

**Lu:** Some of the research themes stem from key development directions of the university and others are linked to the emerging needs of industries. Each topic is selected through iterative consultation and discussion with the professors.

In order to meet the major challenges of science and society nowadays, expertise from one discipline is not enough and interdisciplinary collaboration is required.

—Max Lu

## EXTERNAL PERSPECTIVE ON HIGHER EDUCATION IN CHINA

**NSR:** Compared with the history of higher education in the UK, the history of higher education in China is relatively short. Resumption of college entrance exams in 1977 should be recognized as a milestone in the higher-education history in China.

**Lu:** Yes. Resumption of the college entrance exam has given more people access to higher education and I myself am a beneficiary of this reform. China's higher education has made rapid progress and given rise to many scientific-research achievements in recent decades. If we take high-quality papers published as a reference, many research fields in China have reached the level of highly ranked universities abroad.

**NSR:** There is still dissatisfaction with the speed of science and technology development at home.

**Lu:** The development of science and technology needs foundation and accumulation. Many universities in the USA and UK have histories of over 200 years; their achievements are not achieved overnight. It will take time and patience for China to catch up. It is not exaggerating to say that China is a now big country in science and technology, endowed with high-tech companies like Huawei. It will probably take another 20 years for another 20 ‘Huaweis’ to emerge. At that time, China will be a tech superpower.

**NSR:** Paper publishing is a hot topic frequently discussed in China. Although the publication of articles in the world's top journals does reflect the improvement in the research level of universities and research institutes in China, a large portion of the papers published in foreign journals are considered to be unnecessary and a waste of money.

**Lu:** We should first recognize that papers, as an important output of scientific research and especially of fundamental research, are necessary. Papers may not be ‘useful’ 1 or 2 years after their publication, but they may have a huge impact on technology and the economy 5–10 years, or even decades, later. It is not fair to take ‘usefulness’ as the only criterion for evaluating papers.

But it is also undeniable that, due to the drawbacks of the domestic evaluation system, the phenomenon of counting the number of papers and impact factors of journals is still widespread, resulting in intense pressure on researchers, stimulating the publication of ‘water papers’, which means unimportant papers.

**NSR:** Indeed, in China, large research projects that need interdisciplinary cooperation are likely to become ‘penny projects’: once the fund is allocated, researchers can hardly have substantial collaboration with each other. The underlying reason also relates to the Chinese research-evaluation system.

**Lu:** That is right. If the evaluation of large projects, instead of emphasizing on the first author and primary affiliation, placed more weight on the impact, it would be more favorable for collaborative research.

**NSR:** Interdisciplinary cooperation is more like a mission-driven approach for research.

**Lu:** Yes. The purpose of interdisciplinary research is to meet challenges that cannot be tackled by a single discipline. Such problems are often complex and of great significance. Interdisciplinarity is also an inevitable trend for science advancement and knowledge generation. New frontiers are always emerging at the interface of different disciplines.

**NSR:** How can we solve this problem?

**Lu:** The answer to this question has been long established: establishing the proper and professional research-evaluation system. For instance, for the satellite program I mentioned earlier, its evaluation criteria should clearly be the success of the launch and the realization of designed goals, whereas the publication of papers should not be a requirement. At present, everyone is talking about the Sino–USA trade war, although it brings about a negative effect for both sides, which is also an opportunity for China to meet challenges of sustainable innovation. As for basic research evaluation, peer reviewers should be chosen from all over the world to promote fairness, transparency and quality of assessment.

From my point of view, to mobilize an interdisciplinary team is a daunting task. It requires not only the leadership of strategic scientists, but also the team members who are willing to innovate and dare to break across the disciplinary boundaries. Furthermore, an academic environment in which failure is tolerated and there is a mechanism for punishing academic fraud or misconduct is also an essential prerequisite.

There has been a huge pool of talent and strong investment in scientific research in China; perhaps the most urgent initiative is to establish a more reasonable evaluation system. Since China is getting rid of the traditional evaluating index ‘four only (四唯)’, which focuses on papers, projects, titles and educational background, I think it is a good start.

It is better to improve the research environment rather than having stopgap measures.

—Max Lu

**NSR:** Exploration for better research systems such as national laboratories have been proposed lately. It is known that the US Federally Funded Research and Development Centers (FFRDCs), as the product of the Cold War, established in the 1950s, have been playing a pioneering role in solving the grand challenges of national security and related technology development. They have made breakthroughs in a broad spectrum of fields like information, energy, health and oceans, etc. What can we learn from that?

**Lu:** National laboratories is a top-down design of national science and technology strategy for a country. In China, the national laboratories and their relationship with Chinese Academy of Sciences and universities should be clearly defined. There is no problem in learning from the experience of FFRDCs, but China has its own national conditions. It would be good if national laboratories were planned according to research domains and regions. For example, Beijing national laboratory focuses on AI, Shanghai national laboratory focuses on life science, etc., and they can also address the question of how to mobilize talent and combine the advantages of the region. And to delineate the roadmap around the goal and make a more scientific and fair evaluation system are also important concerns for both government and researchers.

**NSR:** In recent years, some private universities have been established in China, including the Southern University of Science and Technology, Westlake University, etc. What is your opinion on these endeavors?

**Lu:** I think these new types of universities are promising. Southern University of Science and Technology has strong support from the Shenzhen government, the faculty of which have been recruited based on the standard of global renowned universities. The enrollment is also very good, because of their advanced educational concept and rich educational resources.

Westlake University, aiming to be a world-class institute for advanced study, is also located in a place where innovative economy is thriving, facilitating the development of the university. If Westlake University could become a ‘pilot zone’ with the support of the government, to attract world-class faculty members and a global network, I am confident it would be a great success.

**NSR:** There are not many universities with unique features in China at this moment. And it is common to compete with ‘hat talent’ and ranking among universities.

**Lu:** In reality, different institutions have different histories with different characteristics. Like the predecessor of the University of Science and Technology Beijing, Beijing Institute of Iron and Steel Technology, although less known, played an important role in advancing knowledge and innovation in the iron and steel industry in China. Under the existing evaluating system, the homogenization of domestic universities is obvious. Previous specialized institutes have been developing towards comprehensive universities and have gradually lost their unique character and advantages.

**NSR:** Talent draining is common among universities and one of the important motivations is for higher ranking, which leads to talent loss from universities in the underdeveloped western part of China.

**Lu:** Talent mobility is normal and commonplace around the world. I myself was attracted from the University of Queensland to Surrey, and professors from Tsinghua University in China have also been recruited by some famous American universities. But, when it comes to ‘poaching talent’ among domestic colleges, other considerations should be had: for example, what is the deeper reason for talent flowing from the western area to the eastern coast? Is it OK to introduce a policy to prohibit ‘poaching people’? It is understandable that top talent want to work in an environment where they can realize their full potential. In the end, it is better to improve the research environment rather than having stopgap measures. How to retain talent is not only a concern for universities, but also for higher-education administration in China.

## THE FEATURES OF FIRST-CLASS UNIVERSITIES

**NSR:** From your point of view, what are the characteristics of higher education in Austria, the UK and China, respectively?

**Lu:** Australia's higher education is doing well with globalization, with strong competitiveness and extensive academic exchanges. However, the funding mainly comes from the student tuition fees. Due to the narrow industry structure, there is not much support from industry for higher education in Australia.

Higher education in the UK has a strong scientific foundation, good teaching quality, high-level globalization and extensive international academic cooperation. But, compared with the USA, the cultivation of students’ creativity seems to be falling behind and the commercialization of intellectual-property rights is less active. Due to the negative impact of Brexit on the economy and industry, the future of higher education in the UK is uncertain.

‘First-class’ refers to the pioneers instead of followers, who are always leading the world in their fields.

—Max Lu

Higher education in China attaches great importance to mathematics and analytical skills, but pays less attention to diversity and creativity. There are too many evaluation metrics, most of which are directly linked to resource allocation or individual benefits. In fact, there are also evaluation systems in the UK, but the results are often not linked to personal salaries and bonuses. That is one of the major differences between the two systems.

**NSR:** What do you think a first-class university should be like?

**Lu:** Universities are a place where education and research are combined and intertwined. The aim of education is to produce talent to contribute to society. The more contributions the graduates make, the more influence the university would have. Besides education, universities are also a place for research and the creation of knowledge. First-class universities must rely on many first-class disciplines and become prestigious through the long-term accumulation of academic achievements and outstanding culture. ‘First-class’ refers to the pioneers instead of followers, who are always leading the world in their fields.

To sum up, a first-class university should have a first-class management concept, plus first-class financial support as backing; a first-class leadership and management system, plus a first-class culture (excellence, integrity, fairness, transparency and inclusiveness) to attract first-class students and teachers; and a first-class learning and working environment and support, so as to produce first-class alumni, research outcomes and social impact.

**NSR:** Talent and achieving influence are the most direct representations of first-class universities.

**Lu:** Yes. For example, there are several dozens of Nobel Laureates in Cambridge University, which gives rise to the reputation of this university. The cervical-cancer vaccine invented by Professor Ian Frazer at the University of Queensland is an achievement that has had a significant impact on human health. And that kind of invention brings about reputation based on impact for the University of Queensland. A first-class university, boasting either world-class faculty or accomplishments, has obvious advantages.

**NSR:** Diversity is another characteristic of first-class universities.

**Lu:** Yes. A first-class university needs to attract talent worldwide. In Australia, more than 50% of faculty are from abroad. Teachers and students from different cultural backgrounds help to form diversified scientific culture, which is important for sparking new ideas.

## DUTY OF AN EDUCATION LEADER

**NSR:** Can you pursue your research career when you became a president?

**Lu:** It is luxurious to continue your research career when you take the position of president of a Western university. The presidency is a full-time job that requires full-hearted input with no more time to supervise students and continue your research. I have had no laboratory at Surrey since I took the position of president; it is the choice that I had to make.

**NSR:** How did you feel when you decided to be a full-time administrator?

**Lu:** To be honest, it was not an easy decision to become the full-time vice president of the University of Queensland, for I had been enjoying research and doing well. However, I realized that a common goal for both scientists and administrators is to make as great a contribution as possible to society. It is fun to do scientific research, but it also gives me a sense of achievement to lead a university: striving for more resources, making good strategies, and attracting and cultivating excellent talent are essential and a substantial contribution to society.

It is really fantastic that professors can get the support from our university to do the scientific research that they want to do; students can grow and get trained in their studies and will go on to do great things to shape a better society.

**NSR:** The evaluations of a researcher and administer are different.

**Lu:** Right. The purpose of a university is to cultivate talent for society, create new knowledge and make innovations happen. If a university under your leadership could provide the best learning and living experience for its students, offer the best affordable resources and inclusive environment for its professors, I think the president is qualified. And, if people are encouraged to do service to society as appropriate, that is even better. A good president should meet the three requirements and continually make great efforts to improve the quality of the people, work and culture of the university.

I have had no laboratory at Surrey since I took the position of president; it is the choice that I had to make.

—Max Lu

**NSR:** What is your career plan in the next 5–10 years?

**Lu:** My major duty is to bring more resources and attract more talent for the university. We want to grow our scale and research quality. We are going to attract more top-tier professors who can produce first-class research results and/or advance technology transfer to industry. This would not only strengthen our engagement with industry, but also enable graduates to have opportunities to find employment in industry. I look forward to the day when industrial or business sectors will turn to Surrey for solutions whenever they encounter technical and strategical challenges. I also hope that, through our unwavering efforts, the university will be among the top 100 universities in the world within 10 years.

I would also like to address the importance of alumni engagement. As soon as I took office in 2016, I went to China to hold graduation ceremonies and one alumni function, and we set up the alumni associations in China, Singapore, Malaysia and the USA subsequently.

If our alumni achieve more in their career, our university's reputation will be improved and there will be more opportunities that they will give back to their alum mater. It is a virtuous cycle.

**NSR:** What is your suggestion for the young students studying abroad?

**Lu:** More and more young students in China have opportunities to study abroad nowadays; it is important to get a qualification, but it is more important to understand different cultures and learn different ways of thinking, to enrich your life experience. I strongly encourage Chinese students to take a more active part in the students’ activities such as the student union, immersing themselves into the local community and improving social skills. The development of such soft skills is often of vital importance to one's personal development and success in the future.

